# A Popular Guide to the Use of the Bath Waters, with Useful Hints to Visitors; Notes on the Climate of Bath, and Its Advantages as a Health Resort and Place of Residence, &c.

**Published:** 1884-12

**Authors:** 


*A Popular Guide to the Use of the Bath Waters, with useful Hints to Visitors; Notes on the Climate of Bath, and its Advantages as a Health Resort and Place of Residence, &c.* By J. DOUGLAS KERR, Bachelor of Medicine and Master of Surgery, Double Medalist and Hospital Prizeman Glasgow University, Late Resident Surgeon Glasgow Western Infirmary, Consulting Physician on the Bath Waters. Pp. 60. *Bath Herald* Office. 1884.

A fair notion of the purpose of this work will be gathered from its title page, which we copy *verbatim et literatim.* It is slow work wading through its crude and ungrammatical pages; we give our readers the opportunity of joining us in the few smiles its perusal has called forth. One patient, "after a fortnight's bathing, left *with nearly a clean skin.*" The waters are useful in palsy . . . "especially in the *wrist-drop or cholic*, so common among lead-workers and painters." They are also beneficial in "*arthrites*" and in "*leuchorrœa.*" "Hints on Bathing"—"Having procured a ticket at the office beside the Grand Pump Hotel and selected a bath, the invalid will proceed quietly to undress."

That such a work could emanate from any member of our learned profession is, at least, surprising; but that it should come from one of a class whom we have always recognised as among the most cultured and erudite in our profession—the Bath physicians—is most depressing.

